# Multiple Typing Approach to Characterize *Toxoplasma gondii* Strains from Captive and Livestock Species in Northern Italy Suggests the Circulation of Type-II Variants

**DOI:** 10.3390/ani14243597

**Published:** 2024-12-12

**Authors:** Filippo Maria Dini, Martha Ynés Salas-Fajardo, Roberta Taddei, Mattia Ramini, Silvia Vianello, Monica Caffara, Roberta Galuppi

**Affiliations:** 1 Department of Veterinary Medical Sciences (DIMEVET), Alma Mater Studiorum University of Bologna, Via Tolara di Sopra 50, 40064 Ozzano Emilia, BO, Italy; filippomaria.dini@unibo.it (F.M.D.); monica.caffara@unibo.it (M.C.); 2 SALUVET, Department of Animal Health, Faculty of Veterinary, Complutense University of Madrid, 28040 Madrid, Spain; marthays@ucm.com; 3 Istituto Zooprofilattico Sperimentale Della Lombardia e della Emilia-Romagna, Via Bianchi 9, 25124 Brescia, BS, Italy; roberta.taddei@izsler.it (R.T.); mattia.ramini@izsler.it (M.R.); silvia.vianello@izsler.it (S.V.)

**Keywords:** *Toxoplasma gondii*, genotyping, microsatellite, PCR-RFLP, sequencing, mammals, Italy

## Abstract

*Toxoplasma gondii* is a widespread foodborne parasite that affects both humans and animals worldwide. The genetic characterization of this parasite has become crucial due to its epidemiological and clinical implications. The present study focused on the direct genetic characterization of *T. gondii*-positive DNA samples from Northern Italy, using three standardized genotyping methods. Of the 87 PCR-positive DNA samples obtained from livestock, wild, and captive animals, 9 were confirmed, quantified, and subsequently subjected to typing procedures: microsatellite analysis, PCR-RFLP, and partial sequencing of GRA6 and SAG3 genes. Most of the typed samples, derived from clinical cases of toxoplasmosis, specifically related to abortion outbreaks in small ruminants, as well as one case of systemic toxoplasmosis in lemur (*Lemur catta*). Predominantly, Type II strains were observed, with non-clonal variants identified in a sheep and a lemur. Sequencing of SAG3 revealed specific single nucleotide polymorphism, previously documented in European type-II variants, with phylogenesis suggesting a potential genetic relatedness between *T. gondii* population in Italy, Spain, and France. By utilizing three distinct genotyping methods, this study provides the first comprehensive microsatellite profiles of *T. gondii* in Italy, and additional insights into the molecular variability of *T. gondii* strains circulating in this country from different epidemiological settings.

## Simple Summary

*Toxoplasma gondii* is a common parasite that spreads through food and affects both humans and animals worldwide. Understanding the genetic makeup of this parasite is important because it helps us learn more about its spread and effects on health. This study looked at samples from northern Italy to better understand the genetics of *T. gondii* in animals including livestock, wild species, and zoo animals. Out of 87 samples, 9 were further tested using specialized genetic methods. Most cases came from outbreaks of toxoplasmosis—the infection caused by *T. gondii*—that led to miscarriages in sheep and goats, and one case of acute illness found in a lemur. The study mostly identified a specific strain (Type II), but there were unique variants in one sheep and one lemur. Some genetic markers showed links between strains found in Italy, Spain, and France, suggesting a shared genetic background in these regions. This research is the first to provide detailed genetic profiles of *T. gondii* in Italy, giving new information about the variations in this parasite across different animal populations.

## 1. Introduction

The cyst-forming Apicomplexan parasite *Toxoplasma gondii* is known to infect virtually all species of warm-blooded animals. This pathogen poses a significant threat to food safety within the European Union [1] and ranks as the second leading cause of foodborne illness in the United States [1,2,3]. Infections primarily result from the consumption of undercooked meat containing viable tissue cysts or the ingestion of food and water contaminated with oocysts. In humans, these infections are closely associated with harmful effects, such as reproductive failure in pregnant women, neurological manifestations in immunocompromised individuals, and ocular disease in otherwise healthy humans [4].

*Toxoplasma gondii* also has significant implications for animal health, especially in the ovine industry, where it causes abortion and lamb mortality, resulting in substantial global economic losses [5,6,7]. The parasite also has a significant impact on conservation efforts, affecting species highly susceptible to fatal toxoplasmosis, such as lemurs, New World non-human primates (NWNHPs), and Australasian marsupials [8,9,10].

Since the 1990s, researchers have established the existence of three clonal lineages (type I, II, III) distinguished according to their virulence in mice. The development of different molecular techniques has confirmed the predominance of these three clonal/archetypal types or lineages in Europe and North America. However, in some parts of the world, such as South America, frequent recombination events have resulted in a contrasting, extremely diverse and largely non-archetypal population [11]. Genomic diversity within *T. gondii* may significantly influence its epidemiology, affecting factors such as host adaptation in both definitive and intermediate hosts [12,13,14]. Furthermore, some *T. gondii* genotypes are known to exhibit higher virulence towards specific hosts [15,16]. These differences in virulence may exist not only between different host species but also at the intra-host species level [17]. The genotyping of *T. gondii* strains plays a pivotal role in understanding the global population structure of the parasite. There is general agreement that *T. gondii* type II is the most common in Europe, followed by type III. However, a significant proportion of recombinant and atypical genotypes have also been reported, with their phylogenetic classifications still unclear [11]. Moreover, ongoing globalization could create risk situations for the introduction of non-archetypal, highly virulent strains [18,19]. The available genotyping methods, such as PCR-RFLP, PCR–Multilocus Sequencing (MLST) and Microsatellite (MS)-typing, have been inconsistently applied across various geographical regions, using different matrices and methodologies by different research groups [11].

In the last decade, Italy has witnessed the publishing of numerous studies to analyze *T. gondii* isolates to identify prevalent strains (Table 1), often using a varying number of typing loci, which may introduce bias in the characterization of the infecting genotype. This study aims to provide additional insights by analyzing samples collected from both epidemiological surveys and clinical outbreaks of toxoplasmosis in animals from Northern Italy, using three distinct genotyping approaches (MS-typing, PCR-RFLP, and MLST).

## 2. Materials and Methods

### 2.1. Samplig

In this study, we genetically characterized 87 DNA samples of *T. gondii* obtained from epidemiological surveys [46] and routine diagnostic procedures. These samples were obtained from two distinct organizations: The Unit of Parasitology and Mycology, Department of Veterinary Medical Sciences, University of Bologna, and the Istituto Zooprofilattico Sperimentale della Lombardia e dell’Emilia-Romagna. The samples were collected between 2011 and 2023 from the central and north-eastern regions of Italy. The details of the samples included in this study are reported in Table 2.

### 2.2. Parasite Quantification

*Toxoplasma*-specific DNA quantification was performed using a duplex qPCR assay, adapted from Slany et al. [47]. It included the amplification of the species-specific *529RE* locus [48] and an internal amplification control (IAC) to aid the identification of false negative results [49]. The qPCR reactions were performed in a final volume of 25 µL using the SensiFAST Probe Lo-ROX Kit (Bioline, Memphis, TN, USA; BIO-84020); each primer (Tox-9F GTGTTGACGCATCCAGGTCA; Tox-11R CGGATCACTCGTGAACGCTA;) was at a final concentration of 0.25 µM, and HEX (Tox-TP1 HEX-ACCGCCACCGACCAGCACAGC-BHQ, 529RE locus) and Cy5 (Cy5-GGCTCTTCTATGTTCTGACCTTGTTGGA-BHQ, IAC) probes were at a final concentration of 0.15 µM, along with 5 µL of DNA. DNA from clonal type I (TgRH strain), kindly provided by SALUVET group, (UCM, Madrid, Spain) (10 ng/mL) and nuclease-free water were used as positive and negative controls, respectively. Amplification and fluorescence detection were performed on an Applied Biosystems 7500 FAST Real-Time PCR System (Applied Biosystems, Foster City, CA, USA) using 96-well PCR plates under the following conditions: initial denaturation at 95 °C for 5 min, followed by 45 cycles of 95 °C for 15 s and 60 °C for 40 s. Quantification (number of *T. gondii* parasites) was calculated by interpolating the average Ct values on a standard curve equivalent to 1 × 10^5^ – 1 × 10^−1^ tachyzoites generated by tenfold serial dilutions of parasite DNA. Standard curves for *T. gondii* showed an average slope always close to −3.3 and an R2 > 0.98. Parasite load in tissues was expressed as the zoites/mg of tissue.

### 2.3. Microsatellite Analysis

Samples with the lowest Ct values (<36) in duplex qPCR were subjected to genotyping by 15 microsatellite (MS) markers analyzed in a multiplex PCR assay [50]. For *T. gondii* MS typing, we used a set of up to 15 markers located on 11 different chromosomes of the *T. gondii* genome, including eight lineage typing markers (*B18*, *M33*, *TUB2*, *XI.1*, *TgM-A*, *W35*, *IV.1*, and *B17*) and seven fingerprinting markers (*N61*, *M48*, *N83*, *N82*, *N60*, *M102*, and *AA*), that were used to resolve different isolates, applicable to both archetypal (type I, II, or III) and non-archetypal lineages [50]. Following the indications by Joeres et al. [51], the multiplex PCRs were carried out in a final volume of 25 µL, containing 12.5 µL 2X master mix, 2.5 µL Primer Mix (2 µM each primer), 9 µL DNase Free water, and 1 µL DNA. The reaction included positive controls for clonal type I (TgRH), clonal type II (TgMe49), and clonal type III (TgNED) strains, kindly provided by SALUVET group, (UCM, Madrid, Spain) at 5 ng/µL DNA concentration, along with nuclease-free water as a negative control. The cycling conditions were 95 °C for 15 min; 94 °C for 30 s, 61 °C for 3 min and 72 °C for 30 s (35 cycles); and 60 °C for 30 min, using a Veriti thermal cycler (Applied Biosystems, Waltham, MA, USA). Subsequently, PCR products were diluted (1:6) in Hi-Di formamide (Applied Biosystems) and then subjected to capillary electrophoresis with an ABI 3730 DNA Analyzer (Applied Biosystems, Waltham, MA, USA) at the Center for Genomic Technologies of the Complutense University of Madrid (Spain). Fragment size was measured using the bioinformatics software PeakScanner v.1.0 (ABI PRISM, Applied Biosystems, USA) and microsatellite typing results were determined following the guidelines by Joeres et al. [51].

### 2.4. RFLP

Additionally, samples yielding a complete microsatellite profile were subjected to further genotyping analysis. DNA extracts were subjected to the widely used Mn-PCR restriction fragment length polymorphism (RFLP) method, with the markers *SAG1*, *SAG2* (50–30 *SAG2*, and alt. *SAG2*), *SAG3*, *BTUB*, *GRA6*, *c22-8*, *c29-2*, *L358*, *PK1*, and *Apico* [52]. ToxoDB RFLP genotype was identified according to http://toxodb.org/toxo/ (accessed 12 September 2023).

### 2.5. Sequencing and Phylogenetic Analysis

Finally, sequencing of the *SAG3* and *GRA6* markers was conducted for all samples that tested positive in qPCR. These markers are among the most widely available in databases in European context. Sequencing was carried at the Center for Genomic Technologies of the Complutense University of Madrid, Spain, using a BigDye^®^ Terminator kit v 3.1 Applied Biosystems (Foster City, CA, USA). The resulting sequences were assembled with Contig Express (VectorNTI Advance 11 software, Invitrogen, Carlsbad, CA, USA), and the consensus sequences were compared with published data by BLAST tools (https://blast.ncbi.nlm.nih.gov/Blast.cgi, accessed on 9 September 2023). Sequence alignments were carried out by BioEdit 7.2.5 [53]; p-distance and the maximum-likelihood (ML) tree (GTR + G + I substitution model for both genes, bootstrap 1000 replicate) were calculated by MEGA 7 [54].

A phylogenetic tree was constructed based on *SAG3* sequences obtained from our study samples, together with sequences retrieved from GenBank. We selected these supplementary sequences based on their geographical origin, representing Europe and Africa, to contextualize the Mediterranean region. Furthermore, we included sequences from well-established clonal reference strains, such as TgRH (type I), TgMe49 (type II), and TgNED (type III), all sourced from GenBank. All the *SAG3* gene partial sequences generated in this study have been submitted to GenBank under the accession numbers PQ474189–PQ474194.

## 3. Results

### 3.1. Parasite Quantification

Nine out of the 87 DNA samples yielded successful amplification of the 529RE region (Table 3). Notably, the majority of these positive cases (seven out of nine) were derived from domestic small ruminants, all collected during abortion outbreaks. These samples presented parasite loads ranging from 0.35 to 82.5 zoites/mg tissue. Regarding the other two samples, one taken from the muscle tissue of a ring-tailed lemur (acute case) showed a parasitic load of 37 zoites/mg tissue, and the other, from the heart tissue of a red fox, exhibited a burden of 0.3 zoites/mg.

### 3.2. Microsatellite Analysis

Out of the seven samples with a ct value at qPCR < 36, tested with MS-typing, five showed amplification of at least five typing markers. These included three samples from sheep (two placental samples and one CNS sample from a fetus), a muscle sample from a lemur (*Lemur catta*), and a CNS sample from an aborted goat fetus. Two samples (#630: lemur muscle and #1016: sheep placenta) provided a full MS profile with all 15 markers. Consensus profiles were determined for three samples, either having a full profile or at least seven out of eight genotyping markers. These three samples (#1016: sheep placenta, #956: sheep placenta, and #630: lemur muscle) showed non-clonal Type II strains. In Table 4, only the samples with a complete profile are presented. Samples #493 and #268624 were not further analyzed due to the small amounts of DNA.

### 3.3. RFLP

The two samples subjected to PCR-RFLP procedures (#1016, sheep placenta and #630, lemur muscle) showed a complete amplification of all the markers used for PCR and successful enzyme restriction, providing a comprehensive RFLP profiling of *T. gondii*, corresponding to ToxoDB#3 (Table 5).

### 3.4. Sequencing and Phylogenetic Analysis

For subtyping using multilocus PCR-sequencing techniques, we conducted PCR sequencing for two polymorphic genes, GRA6 and SAG3. Readable sequences were obtained from six samples. All GRA6 sequences, corresponding to type II, displayed remarkable (99.7–100%) similarity with sequences from various sources, including dolphin (ON814571, Italy), sheep (MT370491, Spain), and pig (MG587975 and MG587959, Italy), as well as numerous others available in GenBank. Similarly, the SAG3 sequences exhibited a type II allele, though a single nucleotide polymorphism (SNP)-G1691T-was identified, that divided our sequences into two distinct genetic groups. The first group (IIa SAG3 allele), represented by sample #493 (muscle from a congenitally infected goat;), showed 100% matched identity with sequences from different animals and geographical origins, including sheep (MT361125, Spain), cat (KU599489, Turkey), chicken (KU599478, Portugal), dolphin (ON814568, Italy), and the Me49 reference strain (ON814566). The second group (IIb SAG3 allele), characterized by the G1691T SNP, included samples #16, #621, #1016, #956, and #630. This group displayed 100% similarity with sequences from sheep (MT361126, Spain; KU599412 France), cat (KU599488, Turkey), pig (KU599479, Portugal), and dolphin (ON814569, Italy).

**Figure 1:**
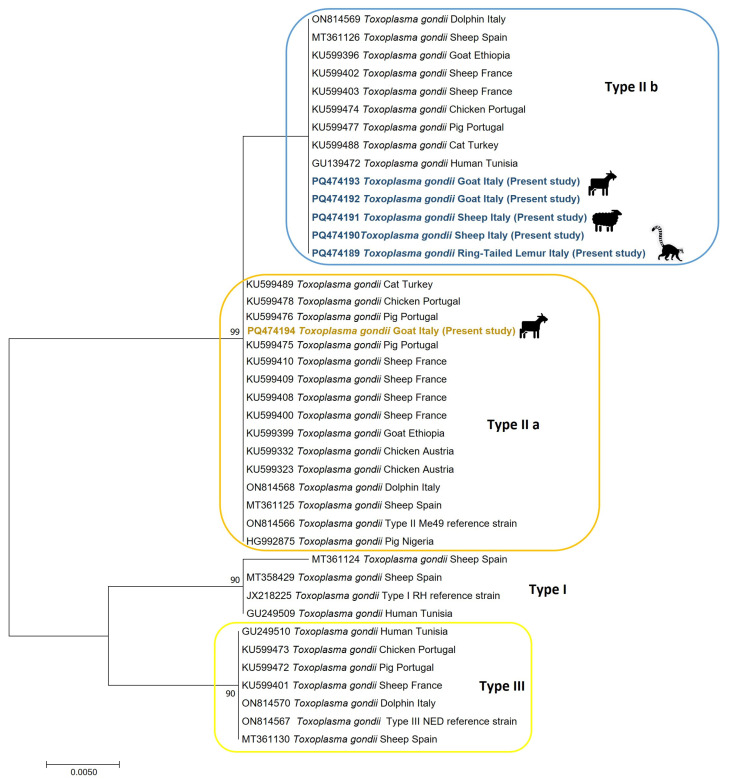
Maximum Likelihood Phylogenetic tree constructed through selected sequences of SAG3 in the Mediterranean context.

The maximum likelihood (ML) (Figure 1) tree revealed three distinct clusters corresponding to Types I, II, and III, with strong bootstrap support values ranging from 90% to 99%. Notably, the SNP G1691T identified in the alignment caused a phylogenetic split within the ML tree, resulting in two distinct subclusters within the Type II cluster. These subclusters effectively distinguish sequences with the G1691T SNP (forming the IIb subcluster) from those representing the classic Type II variant without this mutation.

## 4. Discussion

In this study, we sought to genetically characterize *Toxoplasma gondii* strains isolated from various host species in Italy, collected from both epidemiological surveys and clinical outbreaks, with a particular focus on northern central Italy. Previous studies in this region have reported moderate serological exposure to *T. gondii* in domestic animals and wildlife (dogs [55], wolves and wild boars [56], cattle [57]), as well as molecular evidence in synanthropic micromammals [58]. Notably, a recent serological survey of humans in the same area [59] revealed a 20% seroprevalence in the general population and a 0.4% incidence of acute infections during pregnancy. This evidence underscored the need for increased attention to this significant foodborne pathogen.

The quantitative PCR assay amplifying the *529RE* of *T. gondii* has revealed a substantial reduction in the number of typeable samples. Effective genotyping is typically achieved with high DNA yields, as seen in cases involving bioassay and isolation by cell culture. Studies by Dubey et al. [45] and Fernández-Escobar et al. [23,60], which included a bioassay step prior to genotyping, have yielded more favorable results in terms of marker amplification compared to those that performed PCR-RFLP or MS-typing without a prior bioassay, as observed by Herrmann et al. [61], Calero-Bernal et al. [62], Fernández-Escobar et al. [23], Salas-Fajardo et al. [10], and in the present study. These findings underscore the critical importance of high-quality DNA for obtaining a satisfactory percentage of marker amplification. Achieving type and subtype-level discrimination in *T. gondii*-positive samples can be challenging, especially in epidemiological surveys, where isolation techniques may be both costly and impractical [34]. Moreover, in the wild, samples are often collected days after the animals’ demise, making it difficult to recover viable *T. gondii*. This can result in a lack of harmonization of genotyping techniques across different European countries, making it more difficult to accurately understand the true distribution of circulating genotypes. Specifically, in this study, samples that were frozen after collection were analyzed, and MS-typing and PCR-RFLP results were considered reliable only for those samples displaying complete marker amplification, in order to provide more consistent typing data.

In this study, genetic characterization was achieved using three distinct genotyping methodologies, in order to provide a complete *T. gondii* genetic characterization. Typing results revealed the predominance of Type II variants within our sample set, primarily from sheep and lemur. Type II strains are notably prevalent in European domestic livestock [60,63], wildlife [64], and humans [65]. It is important to note that the current understanding of the genetic diversity of *T. gondii* populations in Italy remains limited. Existing studies often employ a reduced number of typing loci [34,37,41].

Our findings suggest a potential genetic relatedness between *T. gondii* in Italy, Spain, and France. This inference is based on limited *SAG3* sequences of sheep origin available in GenBank. Notably, these sequences are grouped into two distinct clusters, which were delineated by the specific SNP (G1691T), which results in an amino acid change at codon 368 from Methionine (Met) to Isoleucine (Ile), a phenomenon previously documented in a significant number of samples collected from sheep abortion cases in Spain [60] and stranded dolphins in Italy [63].

The exclusive identification of Type II *T. gondii* strains within our collection of small ruminant samples, primarily composed of sheep, aligns with the well-documented literature that underscores the prevalence of *T. gondii* Type II alleles in European sheep populations [5]. *T. gondii* is recognized as a major contributor to ovine reproductive failures, resulting in significant economic losses to the global sheep industry [4,6,7]. It is important to note that all the sheep samples analyzed in this study originated from abortion outbreaks, comprising both placental infected tissue and congenitally infected lambs.

The predominance of Type II *T. gondii* strains among European animals suggests that zoo species in Europe may encounter a relatively restricted set of strain genotypes compared to those found in their natural habitats [66]. Our findings support this trend, as we observed that the single primate specimen in our sample set hosted a Type II variant, clustering in the phylogenetic tree alongside the small ruminant samples displaying the SNP (G1691T), suggesting that the infecting strain is not exotic. In zoological parks, the close proximity of diverse species can facilitate the transmission of pathogens between animals that would rarely, if ever, encounter each other in the wild. This phenomenon was hypothesized by Dini et al. [67] to have occurred in the same zoological park from which the lemur specimens originated.

Lemurs and New World monkeys are especially vulnerable to developing severe clinical manifestations and succumbing to acute toxoplasmosis. While many outbreaks have been reported in captive species, those accompanied by genotypic data remain scarce [68]. In an Italian zoo, a possible type II strain determined by 8 PCR-RFLP markers was confirmed in a ring-tailed lemur (*Lemur catta*) [9]. Lately, another lethal case was reported in a zoo-housed black-capped squirrel monkey (*Saimiri boliviensis*) in Portugal. Genotyping of 13 microsatellite markers confirmed a systemic *T. gondii* infection linked to a type II-like strain [10]. The shared spaces, overlapping enclosures, and sometimes communal feeding areas create potential pathways for transmission, especially among species with similar habitats or behaviors. Such cross-species transmission can pose serious health risks to susceptible animals and may complicate disease management in zoological settings. Monitoring and implementing strict biosecurity measures are essential to prevent outbreaks and protect the health of both the animals and the staff who care for them.

## 5. Conclusions

By employing a combination of three distinct genotyping methods, we have generated the first comprehensive microsatellite profiles for *T. gondii* in Italy. Our findings underscore the predominance of Type II strains, particularly in cases of ovine abortion, and in fatal toxoplasmosis in captive *L. catta*. The results and genetic analysis highlight the importance of using DNA samples originating from diagnostic matrices with high parasitic loads to achieve a comprehensive genetic characterization of *T. gondii*. This multifaceted approach not only enhances our knowledge of *T. gondii*’s genetic variants but also provides valuable insights into its transmission dynamics. Such comprehensive analysis could be beneficial for effectively managing the impact of this pathogen on livestock and captive animal populations.

## Figures and Tables

**Table 1:** Summary of studies reporting *T. gondii* genotypes circulating in vertebrates in Italy.

Region	Host Species	Type (n. Strain Found)	Methods	Isolation	Reference
Liguria	Dolphin (*Stenella coeruleoalba*)	II (1) MRA ^1^ (2)	MLS ^2^ (2 loci)	NO	[20]
Liguria	Dolphin (*Stenella coeruleoalba*)	II (1)	PCR-RFLP (11 loci)	NO	[21]
Tuscan Coastline	Dolphin (*Stenella coeruleoalba*)	II (1)	PCR-RFLP (6 loci)	NO	[22]
Liguria and Southern Italian coasts	Dolphin (*Tursiops truncatus*)	III (1)	PCR-RFLP and seq ^4^	NO	[23]
Liguria and Southern Italian coasts	Dolphin (*Stenella coeruleoalba*)	II (2) III (8) MRA (1)	PCR-RFLP and seq ^4^	NO	[23]
Southern Italy (Cosenza and Napoli)	Dolphin (*S. coeruleoalba*)	III (2)	MS ^3^ analysis	NO	[24]
Sardegna	Sheep (*Ovis aries*—abortion cases)	II (21)	PCR-RFLP (11 loci)	NO	[25]
Toscana	Red Deer (*Cervus elaphus*)	II (1)	PCR-RFLP (12 loci)	NO	[26]
Northern Italy	Sheep (*Ovis aries*—slaughtered)	II (5) MRA	PCR-RFLP (10 loci)	YES	[27]
Apulia	Sheep (*Ovis aries*—milk)	I (1)	PCR-RFLP (1 locus)	NO	[28]
Central Italy	Goat (*Capra hircus*)	II (1)	PCR-RFLP and MLS (1 locus)	NO	[29]
Toscana	Goat (*Capra hircus*)	I (1) III (4) MRA (5)	PCR-RFLP (11 loci)	NO	[30]
Emilia-Romagna	Pig (*Sus scrofa domesticus*)	II (2) MRA (9)	PCR-RFLP (11 loci)	NO	[31]
Emilia-Romagna	Pig (*Sus scrofa domesticus*)	II (2) III (4) MRA (5)	HRM ^5^ and B1-seq	NO	[32]
Lombardia	Pig (*Sus scrofa domesticus*)	I (3) II (4) III (2)	B1-seq	NO	[33]
Piemonte and Lombardia	Wild and domestic mammals (Wild boar, Pig, Cattle, *Vulpes vulpes*, *Roe deer*)	I (31) II (2) III (3) MRA (7)	PCR-RFLP (1–6 loci)	NO	[34]
Piemonte	Alpine Chamois (*Rupicapra rupicapra*)	II (1)	PCR-RFLP (2 loci)	NO	[35]
Piemonte	Wild Boar (*Sus scrofa*)	II (2)	MLS (2 loci)	NO	[36]
Campania	Wild Boar (*Sus scrofa*)	II (1) III (1) MRA (9)	MS analysis (11 loci)	NO	[37]
Toscana	Fox (*Vulpes vulpes*)	I (1) MRA (7)	PCR-RFLP (11 loci)	NO	[38]
Liguria	Horse (*Equus ferus caballus*-slaughtered)	I (1) III (1) MRA (1)	PCR-RFLP (8 loci)	NO	[39]
Toscana	Donkey Milk (*Equus asinus*)	II (1) III (5)	PCR-RFLP (10 loci)	NO	[40]
Toscana	Corvids	II (8) III (7)	PCR-RFLP (5 loci)	NO	[41]
Campania	Eurasian otter (*Lutra lutra*)	II (1)	MS (5 loci); PCR-RFLP and MLS (13 loci)	NO	[42]
Toscana and Liguria	Domestic cat	I (7) II (1) III (7)	PCR-RFLP (11 loci)	NO	[43]
Sicilia	Domestic dog	I (1)	PCR-RFLP (2 loci)	NO	[44]
Southern Italy	Chicken (*Gallus gallus*)	II (3)	PCR-RFLP (10 loci)		[45]

^1^ MRA = patterns showing mixed infections, recombinant or atypical; ^2^ MLS = multilocus sequencing; ^3^ MS = microsatellites analysis; ^4^ seq = sequencing, ^5^ HRM = high resolution melting analysis.

**Table 2:** Samples analyzed by qPCR. In the “tissues examined” column in brackets the number of samples is reported if more than one matrix was analyzed. All DNA extractions were performed using Pure Link ^®^ Genomic DNA Mini kit (Invitrogen, Life Technologies, Carlsbad, CA, USA), according to the manufacturer’s protocol.

Animal Species	N Specimen	Tissues Examined (n)	Amount of Tissue for DNA Extraction
Wolf (*Canis lupus italicus*)	7	CNS (7), Heart Tissue (5)	25 mg
Roe deer (*Capreolus capreolus*)	6	Spleen (6)	25 mg
Red fox (*Vulpes vulpes*)	6	Heart (5), Spleen (1)	25 mg
Mole (*Talpa europaea*)	1	CNS (1), Heart (1), Tongue (1)	25 mg
Least weasel (*Mustela nivalis*)	1	CNS (1), Heart (1), Tongue (1)	25 mg
Hedgehog (*Erinaceus europaeus*)	1	CNS (1), Heart (1), Tongue (1)	25 mg
Squirrel (*Sciurus vulgaris*)	2	CNS (2), Heart (2), Tongue (2), Muscle (2)	25 mg
Wild boar (*Sus scrofa*)	2	Muscle (2)	10 g (200 µL of concentrate after Peptic Digestion)
Northern shoveler (*Spatula clypeata*)	3	Heart (3)	25 mg
Eurasian teal (*Anas crecca*)	1	Heart (1)	25 mg
Mallard (*Anas platyrhynchos*)	2	Heart (2)	25 mg
Northern lapwing (*Vanellus vanellus*)	1	Heart (1)	25 mg
*Lemur catta*	1	Muscle (1)	25 mg
Chicken	12	CNS (4) Heart (3) Muscle (8)	25 g, 4 g, 2 g, respectively (200 µL of concentrate after Peptic Digestion)
Cattle	13	Heart Tissue (13)	25 mg
Goat	4	Liver (1), CNS (Aborted Fetuses) (2), Muscle (Aborted Fetus) (1)	25 mg
Sheep	7	CNS (Aborted Fetuses) (2), Placenta (2), Muscles (Aborted Fetuses) (2), Heart Tissue (1)	25 mg

**Table 3:** *T. gondii* quantification at qPCR.

**ID Sample**	**Specimen**	**Locality of Collection**	**Tissue**	**Ct Value**	**Zoites/mg Tissue**
#630	Lemur	Ravenna	Muscle	26.9	37
#956	Sheep	Parma	Placenta	29.7	24
#1011	Sheep	Mantova	CNS fetus	34	1.5
#1016	Sheep	Mantova	Placenta	27.8	82.5
#22235	Sheep	Parma	CNS fetus	35.8	0.5
#621	Goat	Bologna	CNS fetus	33.9	1.6
#16	Goat	Modena	CNS fetus	30.3	16.2
#493	Goat	Forlì-Cesena	Muscle	36.2	0.35
#268624	Red fox	Modena	Heart	36.5	0.3

**Table 4:** Microsatellite (MS) typing profiles results.

		**Microsatellite Alleles**															
**Sample**	**Species**	**N61**	**B18**	**M33**	**M48**	**TUB2**	**N83**	**XI.1**	**N82**	**TgM-A**	**W35**	**IV.1**	**B17**	**N60**	**M102**	**AA**	**MS Genotype**
#1016	Sheep	93	158	169	211	289	310	356	129	207	242	274	336	140	176	263	II
#630	Lemur	97	158	169	219	289	310	356	111	207	242	274	336	140	174	261	II
TgMe49	*	91	158	169	215	289	310	356	111	207	242	274	336	142	174	265	II

* Type II reference strain.

**Table 5:** RFLP-PCR results.

		**Microsatellite Alleles**												
**Sample**	**Species**	**SAG1**	**3SAG2**	**5SAG2**	**altSAG2**	**SAG3**	**BTUB**	**GRA6**	**L358**	**C22-8**	**C29-2**	**PK1**	**Apico**	**ToxoDB#**
#1016	Sheep	II-III	II	II	II	II	II	II	II	II	II	II	I	ToxoDB#3
#630	Lemur	II-III	II	II	II	II	II	II	II	II	II	II	I	ToxoDB#3

## Data Availability

All the data supporting the findings of this study are included in the manuscript. The DNA sequences generated in this study have been deposited on the public database GenBank.
